# Impact of Side Effects on Anemia Therapy Compliance

**DOI:** 10.3390/nu17091485

**Published:** 2025-04-28

**Authors:** Călina Ciont, Raluca Maria Pop, Ligia Pop, Dan Cristian Vodnar, Ionela-Daniela Morariu, Ramona Suharoschi, Oana Lelia Pop

**Affiliations:** 1 Department of Food Science, University of Agricultural Sciences and Veterinary Medicine, 400372 Cluj-Napoca, Romania; calina.ciont@usamvcluj.ro (C.C.); ligia.olar-pop@usamvcluj.ro (L.P.); dan.vodnar@usamvcluj.ro (D.C.V.); ramona.suharoschi@usamvcluj.ro (R.S.); 2 Molecular Nutrition and Proteomics Laboratory, Institute of Life Sciences, University of Agricultural Sciences and Veterinary Medicine, 400372 Cluj-Napoca, Romania; 3 Department of Morpho-Functional Sciences, Pharmacology, Toxicology and Clinical Pharmacology, “Iuliu Haţieganu” University of Medicine and Pharmacy, Victor Babeș, 400012 Cluj-Napoca, Romania; 4 Department of Environmental and Food Chemistry, “Grigore T. Popa” University of Medicine and Pharmacy, 700115 Iasi, Romania; ionela.morariu@umfiasi.ro

**Keywords:** treatment side effects, absorption, anemia, gastrointestinal symptoms, patient adherence, management

## Abstract

Background/Objectives: Iron deficiency anemia is a prevalent hematological condition globally, with treatment often complicated by adverse effects that compromise patient adherence and clinical outcomes. This study investigated the prevalence, severity, and management of side effects associated with anemia treatments among Romanian patients, aiming to identify key factors influencing treatment adherence and patient satisfaction. Methods: A prospective observational cross-sectional study was conducted using a questionnaire distributed to adult patients diagnosed with anemia. Data were collected from 382 participants, covering demographic variables, anemia causes, treatment types, and patient-reported side effects. Results: Of the participants, 45% reported side effects, with a higher prevalence in intravenous (52%) than oral administration (48%). Common side effects included gastrointestinal symptoms (nausea/vomiting, heartburn, abdominal pain) and systemic symptoms (fatigue, headaches). Our analysis revealed that as the patient age increased, the severity of treatment-related side effects also intensified (*p* < 0.01), particularly in gastrointestinal discomfort. Similarly, BMI was a significant predictor (*p* < 0.05), suggesting that metabolic factors play a role in symptom manifestation. Notably, severe side effects were significantly associated with treatment modifications and lower patient satisfaction. Supplements like magnesium and vitamin D3 showed positive effects in mitigating the side effects, whereas probiotics and vitamin C had mixed outcomes. Conclusions: The study highlights the significant burden of side effects in anemia treatment, emphasizing the need for personalized management strategies to improve adherence and clinical outcomes.

## 1. Introduction

Iron deficiency anemia is a hematological condition characterized by decreased hemoglobin levels and red blood cells, which lead to diminished oxygen delivery to the tissues. Common causes include inadequate iron and nutrient intake, excessive iron losses, chronic illness, and increased iron requirements for growth, pregnancy, or endurance training. Worldwide, anemia remains a public health concern, particularly among vulnerable groups such as pregnant women, children, and individuals with chronic illnesses. The World Health Organization reports that about 24.8% (1.62 billion individuals) of the global population is affected by anemia, which accounts for 80,000 deaths annually [1]. Furthermore, according to the Global Burden of Disease statistics, anemia accounted for 35,057,000 disability-adjusted life years [2]. Anemia was associated with increased fatigue, decreased physical performance, and a higher risk of complications, which can lead to dose reductions or interruptions in therapy [3,4]. Consequently, addressing anemia is crucial for optimizing treatment regimens and improving patient outcomes [5,6,7,8]. Recent studies have highlighted the importance of the early identification and management of treatment-related anemia, emphasizing the need for integrated care approaches [9,10,11].

Food iron fortification and pharmacological treatments, including iron supplements, blood transfusions, and erythropoiesis-stimulating agents (ESAs), are the cornerstones of anemia management [8,9,10,12,13]. Ferrous sulfate, ferrous gluconate, and ferrous fumarate, the most frequently utilized oral iron supplement, have been reported to have a high bioavailability and effectively enhance iron status [3,7,14]. A meta-analysis by Vonderheid et al. (2019) [13] evaluated the effects of various iron supplements on iron absorption and status indicators. The results concluded that while all forms of iron (ferrous sulfate, ferrous gluconate, and ferrous fumarate) supplementation can improve the iron status, ferrous sulfate consistently showed a higher bioavailability than the other forms [13]. These findings were supported by Hurrell et al. (2022), who noted that ferrous sulfate was more sensitive to food matrix effects, which can enhance its absorption compared with ferrous fumarate [14]. However, these therapies were often accompanied by side effects such as gastrointestinal discomfort, constipation, nausea, and, in severe cases, allergic reactions [4,5,9,12]. Such adverse effects deterred patient adherence, compromised treatment outcomes, and exacerbated the burden of anemia [8].

For patients with severe anemia, malabsorption syndromes, or intolerance to oral iron, intravenous iron formulations (ferric carboxymaltose, iron sucrose) have been recommended [6,15,16]. A meta-analysis by Powers et al. (2017) [17] validated that ferric carboxymaltose significantly improved hemoglobin levels more rapidly than oral iron in chronic kidney and inflammatory bowel disease. The analysis included data from multiple randomized controlled trials, highlighting a mean increase in hemoglobin of 1.5 g/dL within 4 weeks [17]. Although generally considered safe, transfusions have been reported to carry risks like transfusion-related acute lung injury and allergic reactions. Studies have reported an occurrence rate of approximately 1 in 5000 transfusions [15,18]. Moreover, moderate allergic responses were noted at 4% after platelet transfusions and severe allergic reactions occurred in approximately 0.04% of cases [16]. Additionally, ESAs (epoetin alfa) have shown efficacy in managing anemia, particularly in chronic kidney disease and cancer patients [19,20]. However, Bhavi et al. (2017) highlighted the increased risk of cardiovascular events, thromboembolic complications, and hypertension in patients treated with ESAs [20].

Studies have shown that selecting an appropriate iron formulation should be approached carefully, considering the pharmacokinetic characteristics of treatment and clinical factors [21,22,23]. Oral iron is recommended for mild to moderate iron deficiency anemia and requires specific timing relative to meals to optimize absorption, leading to a prolonged treatment period before achieving iron repletion [24,25]. On the other hand, intravenous (IV) iron formulations offer a markedly different administration profile [26,27]. For instance, ferric carboxymaltose delivered in a single, high-dose (1000 mg of iron) infusion lasting 15–30 min offered a shifted iron repletion that triggered acute infusion-related reactions including transient hypotension and oxidative stress [26]. Meanwhile, iron sucrose was administered in multiple shorter infusions (10–15 min each), which reduced the intensity of any single reaction but increased the cumulative exposure to infusion-related risks due to repeated administration [27,28].

In recent years, many studies have explored combining different anemia treatments with supplements like probiotics, vitamins, and prebiotics, focusing on their potential to mitigate side effects and enhance iron absorption. However, there is still a significant gap in understanding the patient-reported outcomes related to the side effects of anemia treatment, which can greatly influence treatment adherence and the overall health outcomes.

Given these requirements, the present study delved into the prevalence, severity, and management of side effects associated with iron deficiency treatments among Romanian patients. Our aim was to identify key factors influencing patient adherence to treatment, the role of the healthcare provider in intervention, and the effectiveness of current therapeutic approaches.

## 2. Materials and Methods

### 2.1. Research Design

The research was designed as a prospective observational study with a cross-sectional approach, utilizing an online questionnaire distributed via Google Forms (Google LLC, Mountain View, CA, USA). The primary objective was to investigate the underlying causes of anemia within the Romanian population and to identify demographic and socio-economic determinants linked to its prevalence. In this context, the study aimed to pinpoint vulnerable groups through the analysis of (i) demographic variables, (ii) maternal socio-demographic factors, and (iii) behavioral patterns. The second objective evaluated the prevalence, severity, and spectrum of side effects associated with anemia treatments. Finally, the third objective was pursued to elucidate the interplay between the treatment characteristics, patient behaviors, and the clinical management of adverse effects, providing a comprehensive understanding of factors that influence treatment outcomes.

### 2.2. Subjects

The study population consisted of adult patients (aged 18 and above) with a confirmed medical diagnosis of anemia. Participants were recruited through online patient forums, social media groups, and healthcare platforms targeting Romanian communities. Inclusion criteria encompassed individuals diagnosed with anemia, characterized by low serum iron, low serum ferritin, and hemoglobin levels (<11 mg/dL), who were either currently undergoing treatment or had a documented history of pharmacological therapy for anemia. Patients included in this study received either oral or IV iron supplementation according to clinical guidelines and individualized treatment needs. The iron administration was categorized into three dosage levels: (i) low dose (≤100 mg/day (oral) or ≤200 mg/infusion (IV)); (ii) moderate dose (101–200 mg/day (oral) or 201–500 mg/infusion (IV)); and (iii) high dose (>200 mg/day (oral) or >500 mg/infusion (IV)) [29,30].

Exclusion criteria applied to individuals not receiving anemia treatment, those with self-reported anemia without medical confirmation, and patients who had previously experienced side effects from unrelated treatments. These criteria ensured that any adverse effects reported were directly attributable to anemia therapies, minimizing confounding factors related to pre-existing conditions. All enrolled patients were legally capable of providing informed consent for their involvement in the research. Participants were informed about the purpose of the study, and their privacy and confidentiality were rigorously maintained throughout the study. The study was conducted with meticulous adherence to ethical principles in full compliance with the General Data Protection Regulation (GDPR).

### 2.3. Data Collection

Data were collected from June to December 2024. Initially, a pre-testing phase of the questionnaire was constructed by reviewing the existing patient-reported outcome measures related to the side effects and adherence to anemia treatment. The questionnaire was assessed using a pilot sample of 30 anemia patients who were not part of the final study cohort. Participants provided feedback on question comprehensibility, response format, and the overall structure of the questionnaire. The final survey included 31 questions (multiple-choice questions for quantitative analysis and open-ended questions for qualitative insights) organized into five sections. Firstly, demographic variables, such as age, gender, residence type, education level, and regional location within Romania, were required to assess how socioeconomic and lifestyle influenced anemia prevalence. Socioeconomic status was assessed using a composite measure based on four key indicators: (1) educational attainment (categorized as less than high school, technical school, undergraduate, or graduate degree), (2) employment status (employed, self-employed, unemployed, or retired), (3) self-reported household income categorized into quartiles based on national standards, and (4) residential area type (urban vs. rural). Secondly, the questions focused on prior anemia diagnoses, the timeframe since diagnosis, and the primary causes of iron deficiency. The third section collected data on the treatment type, duration, dosage, and supplementary therapies. Section four detailed the prevalence and severity of specific adverse reactions including nausea, abdominal discomfort, fatigue, gastrointestinal symptoms, and their impact on daily life. These side effects were chosen due to their common occurrence in anemic patients and their ease of reporting from the patient’s perspective. The degree of severity of each side effect was defined according to the Common Terminology Criteria for Adverse Events (CTCAE), version 5 [12]. The severity of side effects of anemia treatment was categorized into five levels: grade 1 (mild) involves asymptomatic laboratory findings or mild symptoms that do not require intervention; grade 2 (moderate) includes minimal, local, or non-invasive interventions; grade 3 (severe) refers to disabling symptoms that require or prolong hospitalization (intravenous iron supplementation); grade 4 (life-threatening) encompasses conditions requiring urgent medical interventions (emergency blood transfusions or oxygen therapy); and grade 5 (death) was assigned when the adverse event leads to death directly related to the treatment side effect. Finally, section five explored patient behaviors related to treatment discontinuation, changes in therapy due to side effects, interaction with healthcare providers, and overall satisfaction with the treatment effectiveness. The questionnaire underwent iterative development and was informed by a literature review and input from subject-matter experts in hematology and public health.

### 2.4. Data Analysis

All data analyses were performed using Origin software version 10 (OriginLab Corporation, Northampton, MA, USA). The significance level for all statistical comparisons was set at 95% confidence. Descriptive statistics were used to summarize the participant characteristics including the means, standard deviations, and frequencies. The Shapiro–Wilk test was applied to assess the normality of continuous variables, and Levene’s test was used to check for the homogeneity of variances. For normally distributed data, independent sample *t*-tests and one-way ANOVA were conducted to compare group differences, while the Mann–Whitney U test and Kruskal–Wallis test were used for non-normally distributed data. Pearson’s correlation coefficients were calculated to assess associations between treatment adherence, adverse effects, and demographic factors. Given the number of correlations analyzed, the Bonferroni adjustment was applied. Logistic regression models were employed to identify predictors of treatment adherence and adverse effect severity, adjusting for potential confounders such as age, gender, socio-economic status, and treatment duration. Odds ratios with 95% confidence intervals were reported for all regression analyses.

## 3. Results

### 3.1. Subject Characteristics

The study received questionnaire responses from 394 participants. Of the 394 pairs, 12 were excluded because the patients had not been medically diagnosed with anemia. The demographic characteristics of the remaining 382 subjects are presented in Table 1. The final cohort was predominantly female (96.85%), with 3.14% male participants, reflecting a higher prevalence of diagnosed anemia among women. The overwhelming female predominance aligns with global epidemiological patterns, where iron deficiency anemia is more prevalent among women due to menstrual blood loss, pregnancy-related demands, and potential dietary differences [31,32]. The age distribution showed that most participants (65%) were between 18 and 45 years old, suggesting a heightened susceptibility to anemia within this demographic, possibly influenced by factors such as reproductive health in women of childbearing age.

Regarding educational attainment, a significant proportion of respondents held a graduate degree or higher (40.57%). Urban residents constituted 70.15% of the sample, underscoring the urban–rural divide in healthcare access and health literacy.

The body mass index (BMI) data indicated that the majority of participants had a normal weight (67.80%), while overweight and obesity were present in 24.60% and 3.40% of the cohort, respectively. Recent research has indicated a significant association between obesity and anemia [33,34]. One significant factor was the chronic inflammation associated with obesity, which can lead to increased hepcidin levels, a hormone that regulates iron metabolism [33]. Elevated hepcidin levels can inhibit iron absorption in the gut and limit iron release from stores, contributing to iron deficiency anemia [34,35]. Additionally, obesity can lead to nutritional deficiencies due to poor dietary habits, further exacerbating the risk of anemia [35].

Behavioral factors revealed that nearly half of the participants were smokers (47.64%), and a substantial proportion reported frequent alcohol consumption (23.95%). These lifestyle factors influence iron metabolism and anemia risk through direct effects on nutrient absorption and associations with comorbid conditions that impact hematological health [36].

### 3.2. Anemia Diagnosis Causes

**Figure 1:**
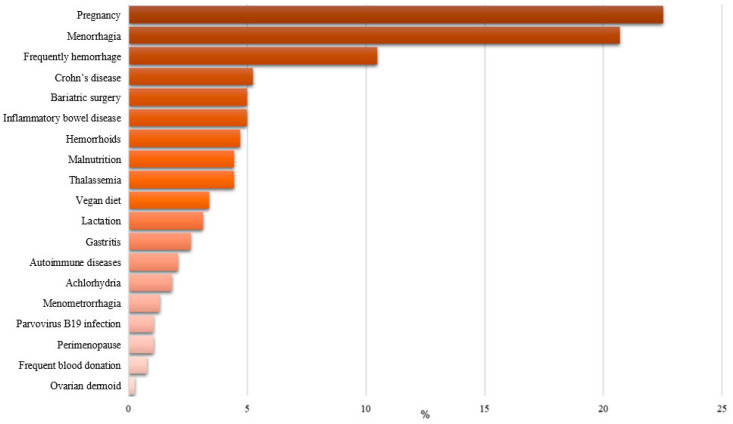
Prevalence of underlying causes of anemia.

The investigation of the causes of anemia diagnosis, as shown in Figure 1, highlights the complex, multifactorial nature of this condition. The most prevalent cause was pregnancy, accounting for nearly 24% of cases, followed closely by menorrhagia (heavy menstrual bleeding) at approximately 21%. The high proportion of pregnancy-related anemia reflects the increased iron demands during gestation, often unmet without adequate supplementation. According to the WHO, anemia affects approximately 38% of pregnant women worldwide, with iron deficiency being the most common cause [1].

Frequently hemorrhage emerged as the third most common cause, suggesting the critical role of chronic and recurrent blood loss in the pathogenesis of anemia. Crohn’s disease and bariatric surgery showed a notable prevalence, highlighting the influence of gastrointestinal disorders and surgical interventions on iron absorption and metabolism. Hazra et al. (2019) found that up to 70% of patients with Crohn’s disease experienced anemia, primarily due to iron deficiency resulting from blood loss and malabsorption [37].

Chronic inflammatory conditions, including inflammatory bowel disease and autoimmune diseases, are significant contributors due to inflammation’s role in disrupting iron homeostasis and erythropoiesis. Similarly, malnutrition and restrictive diets, such as a vegan diet, have been identified as notable causes. Studies have suggested that the inflammation-induced activation of iron regulatory protein 1 plays a significant role in the dysregulation of iron metabolism in IBD, contributing to the progression of anemia [3,5,7]. For these reasons, Indriani et al. (2018) highlighted the role of vitamin D in modulating inflammatory responses, suggesting that deficiencies in this vitamin may exacerbate anemia in chronic inflammatory states [38]. Additionally, vitamin C supplementation (500–1000 mg/daily) reduced inflammatory markers such as hs-CRP and IL-6 in chronic inflammation anemia [39].

Genetic conditions (thalassemia), lifestyle factors (frequent blood donation), and physiological states (lactation, perimenopause) have also been reported but with lower frequency.

These findings underscore the multifactorial nature of anemia, necessitating a comprehensive diagnostic approach that considers both common and less frequent etiologies. The prominence of reproductive health-related causes highlights the need for targeted screening and preventive strategies in women, particularly during pregnancy and their reproductive years. Additionally, the role of chronic diseases and lifestyle factors requires integrated management approaches addressing anemia’s medical and behavioral determinants.

### 3.3. Frequency of Patient-Reported Side Effects

**Figure 2:**
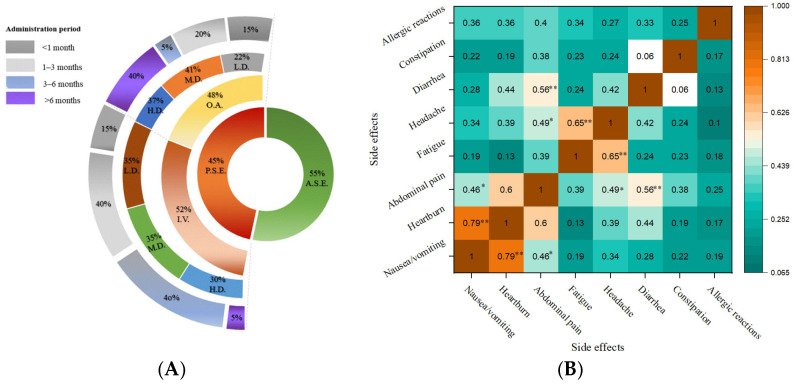
(**A**) Prevalence of side effects of anemia treatment and (**B**) correlation between multiple side effects simultaneously. The data were analyzed using Pearson’s correlation coefficient (r), and the interpretation symbols are as follows: ** *p* < 0.01, * *p* < 0.05. P.S.E.—presence of side effects; A.S.E.—absence of side effects; O.A.—oral administration; I.V.—intravenous administration; L.D.—low dose; M.D.—moderate dose; H.D.—high dose. The iron administration was categorized into three dosage levels: (i) low dose (≤100 mg/day (oral) or ≤200 mg/infusion (IV)); (ii) moderate dose (101–200 mg/day (oral) or 201–500 mg/infusion (IV)); and (iii) high dose (>200 mg/day (oral) or >500 mg/infusion (IV)).

The prevalence of side effects associated with anemia treatments is presented in Figure 2A. Although anemia therapies effectively restore the hemoglobin levels and improve patient outcomes, they are often accompanied by adverse effects that can compromise treatment adherence and overall quality of life [31,37]. The present findings highlight a complex interplay between administration routes, treatment durations, dosage, and patient-reported adverse reactions, with 45% of participants experiencing side effects and 55% not reporting any adverse events. This distribution suggests an overall moderate burden of adverse effects, with a slight majority demonstrating tolerance to therapy without major complications. Similar results were reported by Hazra et al. (2019) in anemic women from India, emphasizing that these side effects lead to treatment discontinuation in up to 50% of patients [37]. Furthermore, among the patients reporting side effects, 48% were correlated with oral administration and 52% with intravenous administration. The higher prevalence of adverse events in the intravenous administration group reflects the distinct pharmacokinetic properties of intravenous formulations, which can potentiate acute adverse reactions during early treatment phases. Furthermore, a marked difference in clinical supervision was observed between the two iron administration routes. Specifically, only about 33% of patients on oral iron discussed their side effects with medical staff during follow-up, whereas all patients receiving IV iron were closely monitored during their infusions. This supervised IV setting likely contributed to a more accurate and timely reporting of acute side effects, allowing for immediate intervention in the case of infusion-related reactions.

Agarwal et al. (2015) [31] reported that IV iron therapy was associated with a higher risk of infections and cardiovascular complications despite more significant increases in transferrin saturation and serum ferritin concentrations than oral iron. The elevated iron saturation in the IV group promoted free iron generation, leading to oxidative stress, endothelial dysfunction, and potentially accelerating atherosclerosis [31]. In contrast, although oral administration generally results in less severe side effects due to slower absorption rates and lower peak concentrations, prolonged use leads to issues concerning gastrointestinal tolerability. Moreover, the results indicate that higher doses of oral iron are associated with a significant increase in gastrointestinal side effects. These adverse effects have been primarily attributed to unabsorbed iron reaching the colon, which interacts with the gut microbiota, leading to oxidative stress, inflammation, and alterations in microbial composition [3,40]. Gastrointestinal symptoms persisted but were less frequent at moderate doses, while adverse events were minimal at low doses. Similar results were reported for high IV doses, which may be linked to the rapid release of iron into the circulation and transient formation of non-transferrin-bound iron (NTBI). NTBI promotes oxidative stress, triggering inflammatory responses and endothelial dysfunction [41]. Adverse reactions were still present at moderate IV doses but at a reduced frequency, whereas low-dose IV administration was associated with minimal side effects. These findings underscore the need for personalized iron therapy strategies, where the choice of administration route and dosage should be tailored to patient-specific factors such as underlying conditions, iron deficiency severity, and tolerance levels [42].

The incidence of adverse effects in IV also rises substantially within the first three months of medication (40% for both <1 month and 1–3 months), followed by a significant decline beyond six months (5%). This pattern suggests an initial phase of heightened susceptibility, potentially due to dose-related toxicity and immunological responses that attenuate as the patient adapts and discontinues the treatment due to intolerability. It has been well-established that most severe IV iron-related adverse events typically occur immediately after infusion [27,43]. These reactions have been primarily attributed to complement activation-related pseudo-allergy and the rapid formation of NTBI [27]. Multiple studies have documented the incidence and management strategies of IV iron-related adverse events, underscoring the importance of proper patient selection, premedication, and adherence to the recommended infusion rates to minimize risks [27,43,44]. The results of Li et al. (2019) [42] indicated that specific dynamic IV strategies in hemodialysis patients led to varying incidences of adverse events, with higher iron indices correlating with increased risks. This elevated risk was especially pertinent with iron overload condition, reinforcing the necessity for a careful assessment of the iron indices prior to IV iron therapy [42]. On the other hand, the side effects associated with oral administration exhibited a more persistent distribution across time. The 1–3-month interval showed the highest prevalence (40%), with a notable proportion of side effects persisting beyond six months (15%). Ghamri et al. (2024) correlated this persistence with continuous gastrointestinal exposure, fluctuating absorption rates, and cumulative drug effects, particularly in individuals with predisposing factors such as gastrointestinal sensitivity [23]. Specifically, ferrous sulfate has been shown to induce dose-dependent digestive issues including nausea, constipation, and mucosal irritation [22,23]. In a randomized controlled trial involving patients with inflammatory bowel disease, the efficacy and safety of intravenous ferumoxytol (510 mg × 2) were compared with oral ferrous sulfate for the 5-week treatment period. The results demonstrated that both therapies effectively elevated the hemoglobin levels, but patients administered oral ferrous sulfate experienced continuous gastrointestinal discomfort during treatment [22].

Furthermore, the administration schedule of iron treatment has been reported to significantly influence the occurrence and severity of side effects [23,45]. For oral iron, alternate-day dosing has been shown to reduce gastrointestinal side effects, such as nausea, constipation, and abdominal discomfort, compared with daily dosing while maintaining comparable efficacy in improving the iron stores and hemoglobin levels [46]. On the other hand, IV iron generally bypasses gastrointestinal side effects but introduces risks of infusion-related reactions (headaches) [47]. Additionally, slower infusion rates have been suggested to mitigate severe reactions [27]. Thus, tailoring the administration schedule based on patient tolerance and clinical needs can optimize efficacy while minimizing adverse effects.

Furthermore, the analysis of patients who reported multiple side effects simultaneously revealed several statistically significant correlations (Figure 2B). In particular, nausea/vomiting showed a strong positive correlation with heartburn (*p* < 0.01), suggesting a shared underlying mechanism. Previous studies have indicated that oral iron preparations increased gastric acid secretion and irritated the gastric mucosa [3,48]. This irritation leads to increased acid production and precipitates heartburn, while irritation of the gastric lining can stimulate the chemoreceptor trigger zone and vagal pathways, inducing nausea and vomiting [48]. Additionally, delayed gastric emptying exacerbated nausea and heartburn due to prolonged exposure of the esophageal and gastric mucosa to acidic contents [3].

Abdominal pain showed significant correlations with both fatigue (*p* < 0.05) and diarrhea (*p* < 0.01). The association between abdominal discomfort and fatigue may be attributable to systemic inflammatory responses and the metabolic burden of persistent gastrointestinal distress. High-dose iron therapy induces significant irritation of the gastrointestinal tract. Data from two different cohorts of the Study of Health in Pomerania revealed that 30% expressed weariness, 16% reported a lack of energy, 16% reported a loss of focus, and 29% reported dyspnea and/or weakness following oral therapy for anemia [49]. Moreover, Fan et al. (2021) found a positive correlation between the inflammatory mediators (IL-6, TNF-α), which contributed to abdominal pain through sensitization of visceral afferent neurons and caused fatigue due to their effects on the central nervous system [50]. Similarly, headache was significantly correlated with fatigue (*p* < 0.05), which aligns with clinical observations where systemic iron overload, dehydration, and medication side effects contributed to both symptoms [32,51]. Interestingly, constipation and allergic reactions appeared relatively independent, with minimal significant correlations, suggesting a more isolated pathophysiological profile.

Collectively, these findings emphasize the importance of a personalized approach to anemia management, wherein the selection of treatment modality is guided by a comprehensive assessment of efficacy, adverse effect profiles, and patient-specific factors to enhance therapeutic adherence and clinical outcomes.

### 3.4. Severity of Patient-Reported Side Effects

**Figure 3:**
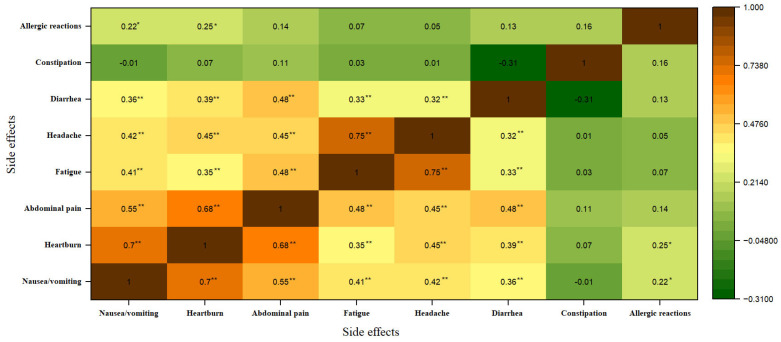
Severity heatmap correlation of the symptoms of adverse effects. The data were analyzed using Pearson’s correlation coefficient (r), and the interpretation symbols are as follows: ** *p* < 0.01, * *p* < 0.05.

The side effects of anemia therapy can range in severity including gastrointestinal difficulties, systemic symptoms, and allergic responses. Figure 3 illustrates the interrelationship between the severity of each patient-reported side effect symptom.

Significant positive correlations were observed between several gastrointestinal symptoms. For instance, the relationship between nausea/vomiting and heartburn (r = 0.70) as well as nausea/vomiting and abdominal pain (r = 0.65) indicates that these symptoms frequently co-occur with high severity (grade 3). Furthermore, heartburn exhibited a strong positive correlation with abdominal pain (r = 0.68), which could be attributed to gastroesophageal reflux disease. Gastrointestinal symptoms often involve the enteric nervous system, which communicates with the central nervous system via the gut-–rain axis [52]. The enteric nervous system communicates with the central nervous system, and disturbances in this communication can exacerbate gastrointestinal symptoms during anemia treatment [52,53]. For example, vitamin B12 deficiency, often in patients with pernicious anemia, leads to fatigue and cognitive disturbances [53]. The severity of these symptoms was associated with the degree of cobalamin depletion, affecting both hematopoietic and neurological functions [54,55].

Conversely, the relationship between abdominal pain and fatigue/diarrhea indicates partial symptom overlap, where one symptom’s severity moderately predicts another’s severity. Researchers have connected this fact with the systemic burden of chronic gastrointestinal disorders, where prolonged inflammation and discomfort trigger a chronic stress response, depleting physical and mental energy reserves. This systemic stress alters the hypothalamic–pituitary–adrenal axis, contributing to persistent fatigue alongside gastrointestinal symptoms. Specifically, patients with inflammatory bowel disease often experience heightened levels of psychological distress, which intensifies abdominal pain and fatigue [21].

**Figure 4:**
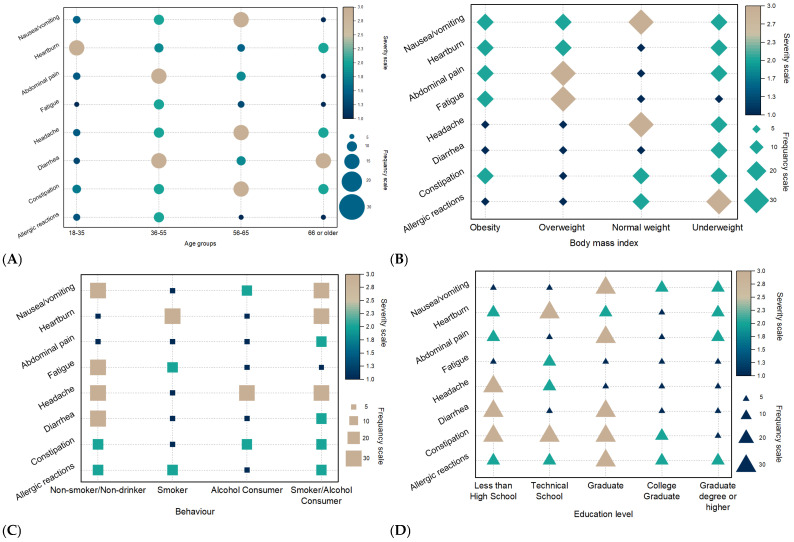
Associations between severity, frequency of side effects, and age period (**A**), body mass index (**B**), behavior (**C**), and education level (**D**). All data are presented as the mean of severity for each side effect.

Furthermore, the severity of side effects induced by anemia treatment was associated with different demographic factors. Figure 4A represents the correlation between side effects and age. Particularly, the severity of nausea/vomiting was more pronounced in the 56–65 and 66 or older age groups, indicating higher severity scores (~2.8). This trend suggests an increased susceptibility to gastrointestinal disturbances, potentially due to age-related drug metabolism and physiology changes. These groups also experienced a higher severity of systemic symptoms (headaches and fatigue) than younger groups, possibly reflecting cumulative health conditions and medication use. In contrast, allergic reactions remained mild across all age groups, indicating minimal age-related variation. Comparatively, younger adults (18–35) reported the lowest severity for most side effects.

Figure 4B depicts the relationship between the severity of patient-reported side effects and BMI categories (obesity, overweight, normal weight, underweight). Individuals with normal weight exhibited higher severity for abdominal pain and headaches, suggesting a potential heightened sensitivity and metabolic response in this group. Underweight individuals reported severe allergic reactions and constipation. Conversely, obese and overweight individuals experienced moderate severity for symptoms like nausea/vomiting and fatigue but displayed a lower severity for allergic reactions. This phenomenon suggests that metabolic factors associated with obesity, such as increased hepcidin levels, protect against certain adverse reactions to anemia treatments [33]. Overall, the severity of side effects appears to vary with BMI, showing heightened vulnerability to specific adverse events.

The association between patient-reported side effect severity and behavior patterns, including non-smoker/non-drinker, smoker, alcohol consumer, and smoker/alcohol consumer, was investigated (Figure 4C). Non-smokers/non-drinkers reported the highest severity for a range of side effects, such as nausea/vomiting, abdominal pain, and headaches, reflecting lower physiological tolerance to external stressors compared with individuals with habitual exposure to such substances. Instead, smokers and alcohol consumers exhibited moderate severity for symptoms (fatigue and constipation). Interestingly, individuals who both smoked and consumed alcohol reported a high severity of gastrointestinal symptoms and allergic reactions, indicating a possible synergistic effect of combined substance use on immune and digestive system sensitivity.

Regarding the education level, the results indicate that individuals with less than a high school education reported the highest severity for multiple symptoms including nausea/vomiting, heartburn, and constipation. Technical school and graduate-level individuals exhibited a moderate severity for symptoms, suggesting a mixed influence of occupational stress and health awareness. In contrast, those with a college degree or higher reported the lowest severity for most side effects, reflecting better health management, lifestyle choices, and preventive care practices. However, individuals with higher education levels may underreport symptoms due to better coping mechanisms, health literacy, or social desirability bias. Also, those with lower education levels might overreport due to heightened health concerns or less familiarity with symptom management strategies. This potential bias highlights the inherent challenges in accurately assessing side effect severity across educational backgrounds, emphasizing the necessity for clinical assessments with self-reported data to mitigate subjective discrepancies and enhance the reliability of health outcome evaluations.

### 3.5. Treatment Satisfaction and Healthcare Interaction

**Figure 5:**
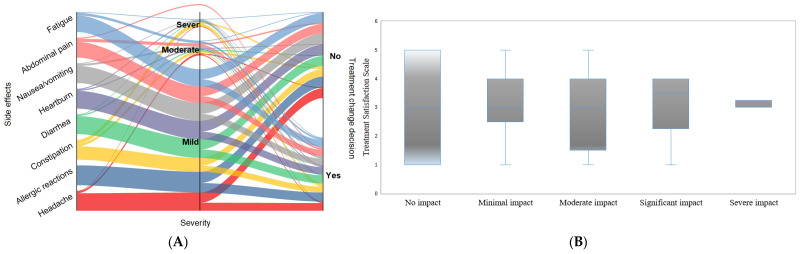
Impact of side effect severity on clinical decision-making and treatment modifications (**A**) and treatment satisfaction in relation to quality of life (**B**). The Sankey diagram (**A**) visualizes the relationship between different side effects, their severity (mild, moderate, severe), and whether treatment modifications were made (Yes or No). The width of each flow represents the proportion of patients experiencing a particular side effect at each severity level and the corresponding treatment decision. The boxplots (**B**) present the variation of the investigated parameters where the midline represents the median values. At the same time, the extreme lines indicate the value of the first and third quartiles.

The subjective experience of side effects, particularly their perceived severity, plays a crucial role in shaping patient satisfaction and influencing clinical decisions regarding treatment modifications [56]. The current findings indicate that severe side effects (fatigue, abdominal pain, and nausea/vomiting) are strongly associated with treatment modifications. Figure 5A illustrates the influence of side effect severity on treatment modifications using a Sankey diagram. The results showed that patients experiencing severe symptoms, such as fatigue, abdominal pain, and nausea/vomiting, were significantly more likely to undergo treatment adjustments including dose reduction or therapy discontinuation. Mild side effects, including allergic reactions and constipation, predominantly correlated with no changes in treatment. Interestingly, moderate side effects showed a mixed pattern, with varying treatment decisions depending on the specific side effect. Patients experiencing significant fatigue often report a decreased quality of life, which can lead to dissatisfaction with their treatment regimen [49]. Iketani et al. (2017) proposed that dose adjustments, particularly in the context of ribavirin therapy, could effectively reduce the withdrawal treatment rates associated with treatment due to side effects such as fatigue and hemolysis [57]. In addition, studies have found that withdrawal treatment rates can be as high as 50% in some populations, particularly when adverse effects are not adequately managed [56,58]. These results highlight the subjective nature of treatment satisfaction, where both the type and perceived impact of a side effect influence healthcare interactions.

Additionally, individuals reporting no impact from the side effects of anemia exhibited the widest interquartile range, suggesting a broad variability in satisfaction levels within this group (Figure 5B). This distribution implies that treatment satisfaction can be influenced by other factors such as personal expectations, comorbid conditions, or healthcare interactions, even without anemia-related complications. On the other hand, groups reporting minimal, moderate, and significant impact showed progressively narrower, widest interquartile ranges with more centralized medians, denoting a convergence of satisfaction levels around the median value. This trend suggests that patient satisfaction becomes more uniform as the side effects become more pronounced. The severe impact category displayed a negative influence of severe anemia side effects on treatment satisfaction, suggesting that such symptoms act as a critical threshold beyond which patients experience converge in dissatisfaction.

**Figure 6:**
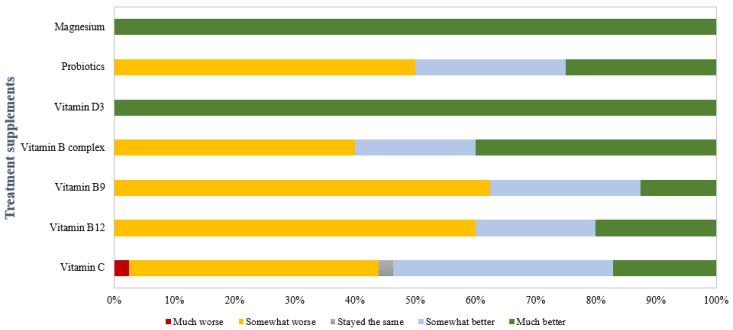
Effectiveness of treatment supplements in reducing the side effects of anemia treatment.

Due to the side effects of anemia treatment, dietary supplements have become increasingly prevalent among patients seeking to enhance their health and manage various conditions [21,23,52]. Figure 6 presents data on the perceived impact of different supplements on reducing the side effects of anemia treatment. Positive feedback was recorded for magnesium and vitamin D3, potentially due to their roles in muscle function, energy metabolism, and immune modulation [59]. Magnesium has a vital role in muscle function and energy metabolism, and its supplementation may help alleviate muscle cramps and improve overall well-being, which can benefit patients experiencing discomfort from iron supplementation [60]. Furthermore, magnesium has been shown to have a laxative effect, which could counteract the constipating effects of iron supplements [60,61]. Similarly, researchers have indicated that vitamin D3 can influence iron metabolism and erythropoiesis [62,63]. A study by Kim et al. (2016) demonstrated a significant association between vitamin D deficiency and anemia in patients with end-stage renal disease [59]. Additionally, vitamin D3 has been shown to lower hepcidin levels, enhancing iron absorption from the gut, thereby improving iron status and alleviating anemia-related symptoms [62,63].

Probiotics exhibited a mixed response, with approximately 40% reporting “somewhat worse” outcomes. However, a substantial proportion noted improvements, indicating variability in individual responses. This heterogeneity may be influenced by the host-specific composition of the gut microbiota, variations in probiotic strains, and the individual’s baseline gastrointestinal health. The negative responses could be related to bloating and gas during the initial stages of probiotic introduction. The responses for vitamin C were also particularly diverse, which could be attributed to gastrointestinal side effects (diarrhea or abdominal cramps) associated with high doses of vitamin C due to its osmotic effect [64]. Additionally, excessive vitamin C intake may exacerbate conditions like kidney stones in susceptible individuals [65].

For the vitamin B complex, vitamin B9, and vitamin B12, most respondents indicated that their health “stayed the same”, with a considerable percentage experiencing “somewhat better effects”.

Overall, the data highlight the heterogeneity in patient experiences with supplements. A more nuanced understanding of the mechanisms underlying these variations could inform tailored healthcare strategies, ultimately enhancing treatment satisfaction and clinical outcomes.

## 4. Limitations

This study’s findings should be interpreted with consideration of its limitations. First, the reliance on data through an online questionnaire may have introduced recall bias, reporting inaccuracies, and social desirability bias. Second, the cross-sectional design restricted the ability to establish causal relationships between demographic variables, treatment modalities, and adverse effects, limiting the inference of longitudinal trends. Income data, in particular, were also categorized based on national quartiles, which may not have fully captured the economic disparities within specific subpopulations. Future studies incorporating objective economic indicators are needed to provide a more comprehensive understanding of the role of socioeconomic factors in anemia treatment adherence and side effects. Third, the sample predominantly consisted of female participants, which may limit the generalizability of the findings to male populations, particularly given the gender-related physiological differences in iron metabolism. Also, potential confounding factors such as comorbidities, concurrent medications, and genetic predispositions were not fully controlled, which may have influenced the reported side effects and adherence behaviors. The lower rate of clinical consultation among oral iron users introduced an element of subjectivity and potential recall bias in the self-reported data, which may have underestimated or overestimated the incidence and severity of the side effects. Finally, the administration schedules were not recorded, preventing a detailed analysis of their impact on side effect prevalence. These findings highlight the need for integrating more comprehensive clinical evaluations in studies of oral iron therapy to align its monitoring practices with those of IV administration and thereby improve the overall robustness of adverse event reporting.

## 5. Conclusions

The findings from this study revealed a significant burden of side effects associated with anemia treatments, which directly impact patient adherence, satisfaction, and overall clinical outcomes. The correlation between adverse effects and the demographic, behavioral, and clinical factors emphasizes the need for a personalized approach to anemia management. Integrating patient education, regular monitoring, and proactive management of side effects could enhance treatment adherence and efficacy. Additionally, healthcare providers should consider demographic disparities when designing intervention strategies to ensure equitable care. Future longitudinal studies with clinical validation are recommended to establish causal relationships and explore the efficacy of adjunct therapies in mitigating treatment-related adverse effects.

## Figures and Tables

**Table 1:** Demographic variables of the participants.

Demographic Variable	Characteristics	*n*	%
Gender	Male	12	3.14
	Female	370	96.85
Age group	18–35	134	35.07
	36–55	201	52.61
	56–65	37	9.68
	66 or older	10	2.61
Education	Less than high school	5	1.30
	Technical school	10	2.61
	Graduate	102	26.70
	College graduate	110	26.79
	Graduate degree or higher	155	40.57
Regional location	Rural	114	29.84
	Urban	268	70.15
Region	South	3	0.78
	Southwest	65	17.01
	Southeast	52	13.61
	Northeast	123	32.19
	Northwest	2	0.52
	Center	134	35.07
	West	3	0.78
Socioeconomic status	Lower middle	89	23.29
	Upper middle	293	76.71
Body mass index	Underweight (<18.5)	16	4.18
	Normal weight (18.5–24.9)	259	67.80
	Overweight (25.0–29.9)	94	24.60
	Obesity (≥30.0)	13	3.40
Behavior	Non-smoker/non-drinker	110	28.79
	Smoking	182	47.64
	Alcohol consumption	92	23.95
	Smoker/alcohol consumer	98	25.52

## Data Availability

The raw data supporting the conclusions of this article will be made available by the authors on request.
